# Treatment of rheumatoid arthritis and its outcomes in an aging society: a single-center cohort study in Japan from 2011 to 2020

**DOI:** 10.1186/s13075-022-02883-x

**Published:** 2022-08-09

**Authors:** Wataru Fukuda, Masatoshi Kadoya, Atsushi Omoto, Takuya Yanagida, Yu Isoda, Atsuhiko Sunaga, Hiroaki Kusuoka, Kentaro Ueno, Satoshi Morita, Masataka Kohno, Yutaka Kawahito

**Affiliations:** 1 Center for Rheumatic Disease, Japanese Red Cross Kyoto Daiichi Hospital, 15-749 Honmachi, Higashiyama-ku, Kyoto City, Kyoto, 605-0981 Japan; 2 Inflammation and Immunology, Graduate School of Medical Science, Kyoto Prefectural University of Medicine, 465, Kajii-cho, Kawaramachi-Hirokoji, Kamigyo-ku, Kyoto, 602-8566 Japan; 3 Department of Biomedical Statistics and Bioinformatics, Kyoto University Graduate School of Medicine, Yoshidakonoe-cho, Sakyo-ku, Kyoto-city, Kyoto, 606-8501 Japan

**Keywords:** Rheumatoid arthritis, Elderly, Quality of life, Health Assessment Questionnaire (HAQ)

## Abstract

**Background:**

We conducted a single-center cohort study of rheumatoid arthritis (RA) patients from 2011 to 2020 to understand their real world treatment and outcomes, especially changes in physical function and quality of life (QOL) in elderly patients, including those aged ≥ 80 years.

**Methods:**

For RA patients attending our outpatient clinic, we annually recorded tender and swollen joint counts, laboratory findings, therapeutic drugs, and scores from the Japanese Health Assessment Questionnaire and EuroQoL-5 Dimensions questionnaire. We examined changes in treatment and outcomes over time, by age group, in patients enrolled over a 10-year period, from 2011 to 2020.

**Results:**

One thousand eight hundred thirty RA patients were enrolled and data were recorded once a year, and a total of 9299 patient records were evaluated. The average age of patients increased by 3.7 years during the study period; the patients aged rapidly. Intensive pharmacological treatment was more frequent in younger patients. Disease activity, physical function, and QOL showed improvement in all age groups over the study period. Physical function and QOL showed greater changes with aging, compared with disease activity. This may be due to the effects of accumulated RA damage, disability due to aging, and depression.

**Conclusions:**

Intensive pharmacological treatment contributes to not only control of disease activity but also the improvement of physical activity and QOL, even in elderly patients. Relieving age-related physical impairment and depression may improve the QOL of very elderly RA patients.

## Background

Significant progress has been made since the 1990s in the drug treatment of rheumatoid arthritis (RA); outcomes have improved markedly [1, 2]. Meanwhile, it has been reported that some drugs also improve physical function and quality of life (QOL) [1, 3].

The aging of society, which is progressing globally, has diverse and significant impacts on medicine and public health [4]. Previous studies have reported a greater prevalence of RA in the elderly population, compared with the younger population, and a greater number of elderly-onset RA patients [5–7]. Elderly patients with RA are generally defined as elderly from the age of 60 to 75 years [8]. However, in Japan, the number of RA patients in their 70s and 80s is rapidly increasing [7, 9].

In T2T recommendations, the most important therapeutic goal in the treatment of RA is to maximize long-term, health-related QOL [10]. Physical activity and QOL generally decline with age. However, in RA patients, age-related changes compound the deterioration in physical activity and QOL caused by disease activity [11, 12].

To date, no clinical studies have examined whether advances in drug therapy can control disease activity and improve physical function and QOL in elderly and very elderly RA patients, as well as in younger RA patients.

Over a 10-year period, from 2011 to 2020, we continuously assessed treatment, disease activity, physical function, and QOL. During this time, the number of treatment options increased significantly, including biologics and targeted, synthetic disease-modifying anti-rheumatic drug (DMARD). Biological DMARD (bDMARD) for RA were available, including infliximab, etanercept, adalimumab, tocilizumab, abatacept, golimumab (launched in 2011), and certolizumab-pegol (launched in 2013). Tofacitinib, a targeted synthetic DMARD (tsDMARD), was launched in 2013. Baricitinib, a Janus kinase (JAK) inhibitor, was launched in 2017; others will be launched after 2019. This study examines the impact of the simultaneous advances in therapeutic agents and population aging on the prognosis of patients with RA.

## Methods

We recruited consenting patients attending our rheumatology outpatient clinic from September to December each year from 2011 to 2022 in this cohort. Patients who had been continuously visiting the clinic since the previous year or earlier were registered as a single patient record based on their age at the time of the visit, as were new outpatients.

We recorded their results from the EuroQoL-5 Dimensions (EQ5D) questionnaire [13, 14], the Japanese Health Assessment Questionnaire (JHAQ) [15, 16], visual analog scale, the number of tender and swollen joints, 28-joint Disease Activity Score C-reactive protein (DAS28-CRP) [17], and medications. We retrospectively examined changes over time in four age groups: < 60 years, 60s, 70s, and ≥ 80s.

### Statistical analysis

Patient backgrounds enrolled in each year were summarized. The overall patient population was also summarized by age group. The DAS28-CRP, JHAQ, and EQ5D data were also analyzed using a linear mixed effects model to account for the longitudinal repeated measures. The model assumed random intercepts for patients and included age and year as the fixed effects. We set the significance level at 5% (two-sided) and report two-sided *p* values. Trends in DAS28-CRP, HAQ score, and EQ-5D score are plotted by year and by age group.

### Ethical considerations

This study was approved by the Ethics Committee of Japanese Red Cross Kyoto Daiichi Hospital. Because it is a retrospective, observational study, the written consent of the patients was not required.

## Results

### Annual change in the number of patients by age group

Fewer than 10 patients per years did not consent to the recording of their data; we consider that this had negligible impact. Over a 10-year period, 1830 RA patients were enrolled and data were recorded once a year, and a cumulative total of 9299 patient records were evaluated. There were 2957 patients aged in their 70s, 2619 patients aged in their 60s, 2459 patients younger than 60, and 1264 patients older than 80 years (very elderly). In a comparison between 2011 and 2020, the average age increased (from 63.9 years to 67.6 years), but the percentage of females did not change over the 10 years (mean, 76.7%). At our hospital, the number of outpatients with RA increased annually; however, when we look at the percentage of each age group by year, we observe that those younger than 60 years old and those aged in their 60s accounted for 30.1% and 34.6%, respectively, in 2011 but decreased to 23.8% and 22.9%, respectively, in 2020. RA patients aged in their 70s accounted for 27.3% in 2011 and increased to 36.1% in 2020. The number of RA patients aged in their 80s increased 3.7-fold (from 52 in 2011 to 194 in 2020); the percentage doubled from 8.1% to 17.2% (Table 1).

**Table 1:** Annual changes in RA patient numbers and the therapeutic agents used in our department

		2011	2012	2013	2014	2015	2016	2017	2018	2019	2020	Total
Number		645	766	811	871	964	1011	924	1064	1117	1126	9299
< 60		194	222	220	223	276	284	238	274	260	268	2459
60–69		223	257	269	274	275	281	240	278	264	258	2619
70–79		176	216	232	263	291	313	314	348	398	406	2957
≥ 80		52	71	90	111	122	133	132	164	195	194	1264
Average age (s.d.)		63.9 (12.5)	64.2 (12.7)	64.7 (13.1)	63.8 (13.2)	65.3 (13.7)	65.7 (13.7)	66.8 (13.0)	66.7 (13.7)	68.0 (13.2)	67.7 (13.7)	66.0 (13.4)
Female (%)		499 (77.4)	592 (77.3)	624 (76.9)	661 (75.9)	734 (75.8)	769 (76.0)	712 (77.2)	813 (76.4)	854 (76.5)	871 (77.4)	7129 (76.7)
Duration, months (s.d.)		110 (108)	111 (110)	117 (114)	129 (118)	116 (110)	120 (112)	132 (124)	126 (128)	122 (113)	117 (113)	120 (113)
MTX dose (mg/w)		7.15 (3.88)	7.44 (4.11)	7.49 (4.12)	7.65 (4.19)	7.69 (4.23)	7.70 (4.25)	7.76 (4.61)	7.71 (4.26)	7.6 (4.22)	7.62 (4.20)	7.60 (4.23)
Use (%)		437 (67.8)	501 (65.4)	523 (64.4)	568 (65.2)	608 (63.1)	644 (63.7)	555 (60.1)	585 (55.0)	610 (54.6)	618 (54.9)	5649 (60.6)
Use/dose	< 60	71.6/7.89	73.0/8.15	68.4/8.47	71.3/8.45	66.3/8.58	73.2/8.42	71.8/8.48	70.1/8.52	68.8/8.39	64.6/8.29	69.8/8.38
(%/mg/w)	60–69	68.6/7.26	68.1/7.57	67.6/7.62	70.1/8.02	68.4/8.10	68.3/7.98	65.8/8.05	62.2/8.02	61.4/8.02	67.8/7.95	66.7/7.87
	70–79	65.3/6.55	58.8/6.90	62.9/6.85	61.6/7.03	60.5/7.01	58.5/7.30	56.5/7.25	44.8/7/14	52.8/7.07	52.7/7.21	57.1/7.06
	≥ 80	57.7/5.32	52.1/5.59	47.8/5.67	49.5/5.85	50.0/5.77	45.9/5.54	37.9/6.18	39.0/6.02	30.3/5.95	28.9/6.05	40.5/5.83
PRED dose		4.20 (2.38)	4.06 (2.35)	4.49 (2.71)	4.72 (2.70)	4.56 (2.62)	4.80 (2.78)	4.79 (2.81)	4.48 (2.64)	4.48 (2.65)	4.62 (2.85)	4.53 (2.67)
use (%)		199 (30.8)	220 (28.7)	236 (29.0)	249 (28.6)	274 (28.4)	287 (28.4)	260 (28.1)	263 (24.7)	337 (30.2)	338 (30.0)	2663 (28.6)
bDMARDs (%)		178 (27.6)	170 (22.2)	200 (24.7)	193 (22.2)	199 (20.6)	289 (28.6)	212 (22.9)	256 (24.1)	256 (22.9)	271 (24.1)	2224 (23.9)
TNFi (%)		75.3	68.2	70.0	67.9	65.3	69.9	50.9	52.0	48.8	49.8	60.9
IL6Ri (%)		18.5	21.8	20.5	23.3	26.1	19.0	30.2	26.6	30.5	32.5	25.2
Abatacept (%)		6.2	10.0	9.5	8.8	8.5	11.1	18.9	21.5	20.7	17.7	13.9
tsDMARDs (%)		0 (0.0)	0 (0.0)	0 (0.0)	1 (0.1)	6 (0.6)	11 (1.1)	18 (1.9)	29 (2.7)	46 (4.1)	55 (4.9)	166 (1.8)

*Abbreviations*: *s.d.* standard deviation, *MTX* methotrexate, *PRED* prednisolone, *bDMARDs* biological disease-modifying anti-rheumatic drugs, *TNFi* tumor necrosis factor inhibitor, *IL6Ri* interleukin-6 receptor inhibitor, *tsDMARDs* targeted synthetic disease-modifying anti-rheumatic drugs

### Changes in therapeutic agents over time

In 2011, methotrexate (MTX) was widely used in Japan as an anchor DMARD. Trends in treatment over the 10 years show that the frequency of MTX administration decreased from 67.8%, in 2011, to 60.1%, in 2017, to 54.9%, in 2020. A comparison of the percentage of MTX use by age group shows that the decline mainly reflects a decrease in the elderly, 70 years and older. The average dosage of MTX did not change significantly, remaining constant at 7.44–7.76 mg/week after 2012, when the national dosage limit was relaxed from 8 mg/week to 16 mg/week. The frequency of prednisolone (PRED) administration ranged from 30.8 to 28.6%, and the dose ranged from 4.20 to 4.53 mg/day, both of which did not change significantly over the 10-year period. The frequency of administration of biologics, as a whole, did not change over time. However, there were changes in the type of biologics administered. The use of tumor necrosis factor (TNF) α inhibitors decreased from 75.3 to 49.8%, while the use of interleukin (IL) 6 receptor inhibitors and of abatacept increased. In 2020, tsDMARDs were administered to 55 patients (4.9%) (Table 1).

### Comparison of therapeutic drugs by age

The average frequency of MTX administration over 10 years was 60.6% and the average dose was 7.60 mg/week. Both frequency and dosage tended to decrease in elderly patients, with only 5.13 mg/week being administered to 40.8% of patients over 80 years of age. PRED was administered to 20.6% of patients aged < 60 years, which increased to 51.3% in patients aged ≥ 80 years; there was no difference in dose between the age groups. The frequency of biologics administration decreased with age. IL6 receptor inhibitors, in particular, accounted for 27.5% of all biologics administered in patients aged < 70 years but decreased markedly to 5% in patients aged ≥ 80 years. The proportion of patients receiving abatacept increased in elderly patients, accounting for 21.0% of all biologics in patients aged ≥ 70 years, and 36.8% of all biologics in patients aged ≥ 80 years. There was no difference between age groups in the frequency and type of tsDMARD administered (Table 2).

**Table 2:** Changes in the therapeutic agents used, by age group

	<60	60–69	70–79	≥ 80	Total
Number	2459	2619	2957	1264	9299
MTX use (%)	1718 (69.9)	1750 (66.8)	1665 (56.3)	516 (40.8)	5649 (60.6)
Dose (s.d.)	8.38 (4.40)	7.87 (4.36)	7.06 (3.89)	5.13 (3.83)	7.60 (4.23)
PRED use (%)	507 (20.6)	615 (23.5)	892 (30.2)	649 (51.3)	2663 (28.6)
Dose (s.d.)	4.91 (2.52)	4.41 (2.42)	4.42 (2.66)	4.51 (3.16)	4.53 (2.67)
bDMARDs (%)	713 (28.9)	651 (24.8)	633 (21.4)	228 (18.0)	2224 (23.9)
TNFi (%*)	480 (67.3)	418 (64.2)	329 (49.6)	132 (57.8)	1354 (60.9)
IL6Ri (%*)	203 (28.5)	175 (26.9)	171 (27.0)	12 (5.3)	561 (25.2)
Abatacept (%*)	30 (4.2)	58 (8.9)	133 (21.0)	84 (36.8)	309 (13.9)
tsDMARDs (%)	50 (2.0)	31 (1.2)	64 (2.2)	21 (1.7)	166 (1.8)

%* means percentage of the drug in used b/ts DMARDs

*Abbreviations*: *MTX* methotrexate, *s.d.* standard deviation, *PRED* prednisolone, *bDMARDs* biological disease-modifying anti-rheumatic drugs, *TNFi* tumor necrosis factor inhibitor, *IL6Ri* interleukin-6 receptor inhibitor, *tsDMARDs* targeted synthetic disease-modifying anti-rheumatic drugs

### Change in disease activity by age group

**Fig. 1:**
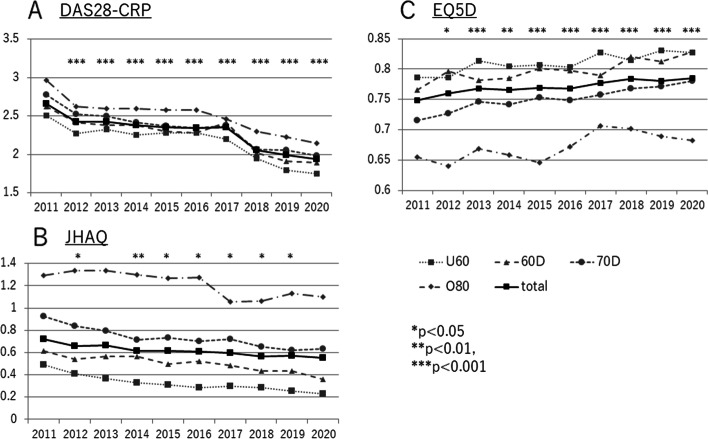
Annual trends and changes in RA treatment outcomes from 2011–2020 for each age group. **A** Annual change of mean DAS28-CRP values, for all patients and for each age group. **B** Annual change of mean JHAQ scores, for all patients and for each age group. **C** Annual change of mean EQ5D values, for all patients and for each age group. Abbreviations: DAS28, 28-joint Disease Activity Score; JHAQ, Japan Health Assessment Questionnaire; EQ5D, EuroQol-5 Dimensions questionnaire

**Fig. 2:**
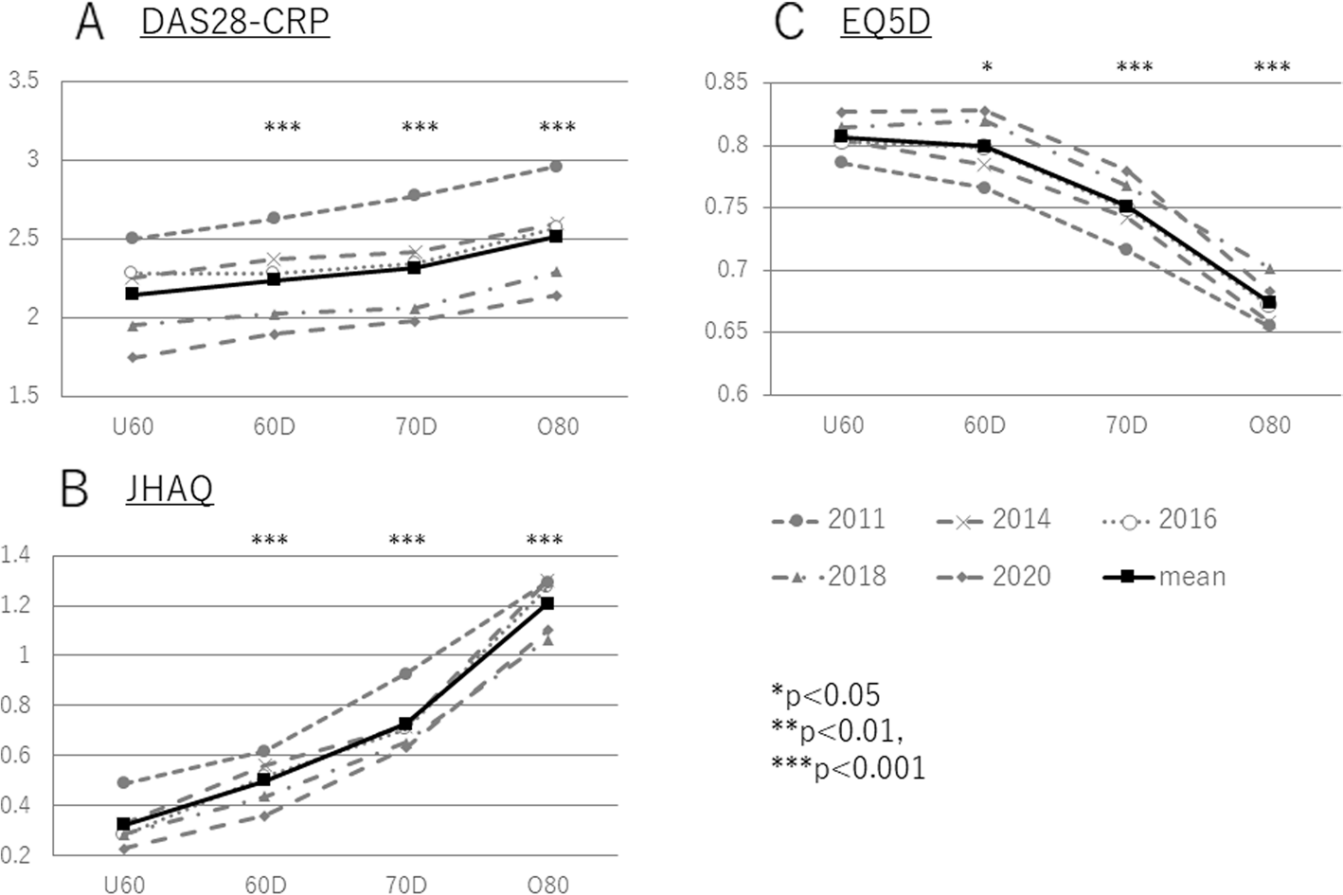
Changes of RA treatment outcomes by age groups. **A** Changes in mean DAS28-CRP values, by age group, for the entire period and for each year. **B** Changes in mean JHAQ scores, by age group, for the entire period and for each year. **C** Changes in mean EQ5D values, by age group, for the entire period and for each year. Abbreviations: DAS28, 28-joint Disease Activity Score; JHAQ, Japan Health Assessment Questionnaire; EQ5D, EuroQol-5 Dimensions questionnaire

The rate of change of DAS28CRP varied from year to year between 2011 and 2020. However, the average for all patients decreased significantly each year, from 2.66 to 1.93 (*p* < 0.001). Similar improvement was shown by all age groups, including the ≥ 80 years group (Fig. 1A). The average DAS28CRP score was lowest in the < 60 years age group, at 2.16. It deteriorated linearly with age, the average score rising to 2.26 (≥ 60 years), to 2.34 (in the 70 years), to 2.51 (≥ 80 years). To consider the magnitude of change in DAS28-CRP, we assigned a value of 1 to the difference between patients aged < 60 years and those aged in their 60s. In terms of this index, the difference between patients aged in their 60s and those aged in their 70s is 0.74. The difference between patients aged in their 70s and those older than 80 years is 1.61 (Fig. 2A).

### Change in JHAQ by age group

The JHAQ score is a measure of physical activity. Annual changes in the average score for all age groups showed a significant improvement (from 0.72 to 0.56) over the past 10 years (*p* < 0.001). Patients aged > 80 years showed an improvement from 1.29 to 1.10 (Fig. 1B). The lowest score was 0.245, for those aged < 60 years. The score increased with age to 0.50 (60s), to 0.73 (70s), to 1.03 (80s and older). For patients aged in their 60s and 70s, the magnitude of change was 1.71 times greater than for younger patients; for older patients, it was 3.48 times greater (Fig. 2B).

### Change in EQ5D by age group

The EQ5D score was chosen as a measure of QOL. Annual change in this score was slow but significant, improving over the 10 years from 0.749 to 0.785 (*p* < 0.001). This trend was similar for all age groups. Although patients aged in their 80s did not necessarily show a linear increase in EQ5D score, there was no difference among the ages when comparing the differences between 2011 and 2020 (Fig. 1C). The mean EQ5D score for each age group differed slightly for those aged in their 60s (0.80), compared with those younger than 60 (0.81). It significantly worsened for those aged in their 70s (0.75) and 80s (0.67) (*p* < 0.001 for each). As was found for the JHAQ score, the magnitude of change in the EQ5D score differed only slightly between patients aged in their 60s and those younger than 60. However, there was a marked deterioration with increasing age. The magnitude of change was 3.9-fold greater for patients aged in their 60s and 70s, and was 6.58-fold greater for patients aged in their 70s and older (Fig. 2C).

## Discussion

Lower treatment efficacy in elderly-onset RA and elderly RA patients, compared with younger patients, has been reported by many studies that have examined therapeutic agents and outcomes. It has been reported that elderly patients have higher risks of adverse reactions, including infections, and lower rates of drug retention [8, 18]. Moreover, most reports define “elderly” as the age range from 60–75 years old. There is little data on the patient population older than 80 years, which is expected to increase in our aging society. Recent advances in drugs and therapeutic strategies have improved the outcomes of RA [1, 2]. However, to date, there is no satisfactory answer to the question of whether, in elderly patients, these advances have outweighed the effects of aging on disease activity control and functional prognosis. Japan is the most aged country in the world [19], and the present cohort study followed treatment options and outcomes for 10 years in a population that included patients older than 80 years. It is expected to provide some answers to the clinical questions raised above.

When considering the change in the numbers of RA patients from 2011 to 2020, two factors need to be considered. First, the Department of Rheumatology was established in our hospital in 2004; the increase in patients since 2010 may have been due to the expansion of the department. Thus, the increased number of patients during this period does not necessarily imply that the number of RA patients in the region increased. Second, our hospital is located in an area with a particularly large elderly population, even by Japanese standards. In Higashiyama Ward, the proportion of residents aged ≥ 65 was estimated to be 32.9% in 2020, compared with 28.6% in Japan as a whole [20]. This implies that the effects of aging shown by this study may be more pronounced in Japan as a whole, where the population is aging rapidly. The average age of the RA patients included in this study increased by 3.8 years over the 10-year period. The annual number of patients aged in their 70s increased from 27.3% to 36.1%; the annual number of patients aged 80 years and older increased from 8.1 to 17.2%, which indicates that the RA patient population is aging rapidly.

In general, it is known that the proportion of males increases in elderly-onset RA patients [8, 18]; in this study, there was no change in the sex ratio. This may be because the increase number of male RA patients was offset by an increase in deaths; the estimated 2020 average life expectancy of males in Japan is 81.64 years, 6.1 years shorter than that of females (87.74 years) [20].

When we examine changes in the choice of therapeutic agents and outcomes over the past 10 years, we believe intensive drug therapy was used whenever possible, taking into account the patient's age and physical and social situation. MTX is said to be an anchor drug for RA. In this study, MTX was the mainstay of RA treatment, used in more than 60% of patients, although at a lower dosage than in Europe and the United States. The frequency of MTX use decreased after the age of 70, especially in patients aged ≥ 80 years (40.8%). The amount of MTX used also decreased. PRED was used more frequently in elderly patients, but the dosage (4.41–4.91 mg/day) did not differ depending on the age. MTX has serious side effects, as previously reported, which are especially to be avoided in elderly patients. It is presumed that low-dose steroids were selected as alternative treatments [8, 21, 22]. The usage of biologics, as a whole, tended to decrease with aging; especially IL6 receptor inhibitors were avoided in patients older than 80s. On the contrary, the use of abatacept increased in very old patients. This may be due to evidence in favor of abatacept, compared with IL6 receptor inhibitors and TNF inhibitors, regarding the risk of severe infection [23].

Disease activity in RA improved almost linearly in all age groups over the 10-year period. Considering that there was no change in all ages with respect to MTX dosage during the 10 years, it is assumed that there was no significant change in the severity of the patients. Therefore, we believe that this is mainly due to the emergence of new therapeutic agents and the penetration of intensive therapeutic strategies. Treatment outcomes by age group showed that disease activity deteriorated almost linearly from the < 60 years group to the ≥ 80 years group. This is probably related to the fact that the frequency of use of highly effective drugs, such as MTX and biologics, decreases with age. However, improvement in disease activity over the past decade was generally similar between patients aged > 80 years and those younger than 60 years, which indicates that advances in intensive drug therapy have improved outcomes in elderly patients and younger patients equally.

Physical function, as assessed by the JHAQ, and QOL, as indicated by EQ5D, also improved in all age groups over the decade. This indicates that tight control of disease activity in RA is useful for improving physical function and QOL in both young and elderly patients. However, the pattern of change by age group for both differed slightly from that of disease activity. The magnitude of change in deterioration increased with age. Several causal factors must be considered. In terms of physical function, it is important to consider the deterioration caused by residual joint destruction, and the irreversible component of a health questionnaire [11, 24]. Joint deformities and contractures caused by RA will persist, even after achieving remission with treatments. Second, age-related changes that are not related to RA, such as osteoarthritis, osteoporosis, and sarcopenia, increase in prevalence with age and contribute to functional impairment [11, 25]. Psychological depression is believed to be a third factor contributing to deterioration of QOL. It is well known that RA patients are prone to depression [26], which is exacerbated by aging and associated physical disabilities [27]. The psychological burden of RA may be increased by age-related changes in the home and social environment. These three problems, which worsen HAQ and QOL in late-stage elderly RA patients, are expected to have an increasing impact. Non-pharmacological approaches, such as physical care and psychological support, should be provided along with pharmacotherapy for these patients [28, 29].

The study has several limitations. The living environment of elderly patients and their treatment choices for RA are influenced by economic, cultural, and political factors; therefore, regional differences are likely to be significant. This study was conducted at a single hospital in Japan for a limited period of time; it may not necessarily apply to different social situations or time periods. Second, this study did not examine several variables that may have a significant impact on physical function and QOL, such as comorbidities and socioeconomic status. How these variables relate to the age and disease activity presented here is a very important question that will have to be examined in the future. Third, the number of physicians who treated the patients was limited; their treatment strategies and drug choices may not have always been standardized. However, it can be inferred from the aforementioned medication details that the RA patients registered in this study were treated intensively to achieve the treatment target.

## Conclusions

With global population aging, the numbers of RA patients with elderly onset and those aged 80 years and older will increase. Our results show that control of disease activity with DMARDs, based on T2T, can contribute to improvements in physical activity and QOL in late-stage elderly patients with RA. However, for improving the QOL of these patients, the effects of accumulated RA damage, disability due to aging, and depression (due to various factors) must be approached as important issues for future RA treatment.

## Data Availability

The datasets used and analyzed during the current study are available from the corresponding author on reasonable request.

## Abbreviations

bDMARDs: Biological disease-modifying anti-rheumatic drugs
DMARD: Disease-modifying anti-rheumatic drug
EQ5D: EuroQoL-5 Dimensions questionnaire
ESR: Erythrocyte sedimentation rate
HAQ: Health Assessment Questionnaire
IL6R: Interleukin-6 receptor
JHAQ: Japanese Health Assessment Questionnaire
MTX: Methotrexate
PRED: Prednisolone
QOL: Quality of life
RA: Rheumatoid arthritis
TNF: Tumor necrosis factor
T2T: Treat to Target
tsDMARDs,: Targeted synthetic disease-modifying anti-rheumatic drugs
